# Supplementary material for: Trends and Factors Associated with Obesity Prevalence in Rural Australian Adults—Comparative Analysis of the Crossroads Studies in Victoria over 15 Years
Source: Nutrients. 2022 Oct 28;14(21):4557. doi: 10.3390/nu14214557 (PMC9659019; doi:10.3390/nu14214557)
Supplement: Supplementary file 1 [file nutrients-14-04557-s001.zip › nutrients-1909202-supplementary.pdf]

Article

Trends and Factors Associated with Obesity Prevalence in Rural Australian Adults - Comparative analysis of the Crossroads Studies in Victoria over 15 years

Supplementary Material:

Table S1. Previous studies on dietary intake in rural and urban Australia. Literature search used the following search terms "diet", "intake", "food", "fruit" and "vegetables", alongside "obesity", "BMI", "waist circumference" and "rural Australia". Studies were assessed and excluded if they targeted a specific gender, ethnicity, or cultural background for the purpose of consistency in cohort comparison.

Authors (Year)	Location	Gender	Sample Size	Limitations	Main Findings	Recommendations
R. Pullen Et al. 2021	Rural & regional New South Wales, Australia (AU)	53% female	247 adults aged 18+	Small sample size. Lack of income information	Rurality did not return a relationship to diet quality in this setting. Living alone a stronger link to diet quality Link between chronic conditions and poor diet quality	Further research of a larger sample from rural/regional setting. Remove grouping of predefined health conditions from future research, allowing more flexibility in investigating diet quality and health outcomes. Effort to be made in assisting those living with chronic condition to improve diet
D. Simmons Et al. 2020	Rural Victoria, AU	~55% female	Crossroads I-5258 Crossroads II-2649	Selection bias in clinic participants. Selection bias in missing lifestyle data. Only one rural region, does not represent all rural areas	Prevalence of diagnosed diabetes increased from 5.0-7.7%. Screening increased. Undiagnosed diabetes decreased	Further work needed to increase uptake of lifestyle changes to reduce prevalence of type 2 diabetes mellitus (T2DM)
P. Love Et al.	Rural Victoria, AU	-	10 rural local government	Seasonal data-conducted in winter.	Confirmation of parallel between	Continued research into the influence of

2018			areas (LGA's) in Victoria.<br>39 retail food outlets	Food environments constantly change.<br>No data collected re consumer venue preferences.<br>No data collected for utilization of community gardens, food swaps, food bank etc.<br>Flaws with Healthy Diets ASAP tool.	food insecurity, low income, and obesity with rural/regional locations.<br>Healthy diet as recommended by National Dietary Guidelines is cheaper than current diet consumed by Australians;<br>Challenge perceptions that eating healthy is more expensive.	food environments among rural communities.<br>Establishing public health campaigns and programs which target improvement of food literacy knowledge and skills.
S. Lim Et al. 2017	Rural Victoria, AU	61% female	1154 adults of Grampians region	Self-reported data.<br>Selection bias.<br>Cross-sectional	Health literacy is associated with greater fruit and vegetable intake	Greater attention paid to health literacy in public health promotion for improved fruit and vegetable intake.
C. Milte Et al. 2015	Urban and rural, Victoria, AU	1150 males 1307 female	Adults aged 55-65	Non-quantifiable FFQ used.<br>No adjustment for energy intake in statistical analysis.<br>Income/financial status not included.<br>2 years between surveys.<br>Self-reported data	Better quality diets resulted in better quality of life.<br>Greater emotional wellbeing observed in women with better diet quality.	Further longitudinal analysis over longer periods of time.<br>Further investigations over time into the impact of diet on quality of life.<br>Greater focus on addressing diet quality in population programs/ policy to support healthy ageing.
N Davis-Lameloise Et al. 2013	Limestone Coast, South Australia; Corangamite Shire & Wimmera Region, Victoria, AU	-	1001 adults aged 25-74	Localised sample.<br>Morbidity and mortality of occupational groups not accounted for	Agricultural workers had healthier diets<br>Male technicians more likely to consume take away food, poorer diet	Increased health promotion to improved diet and physical activity.<br>Targeted programs to occupational groups
E.D. Williams Et al.	Rural Victoria, AU	-	4405 people	Self-reported data bias,	Correlation between level of education and	Development of health promotion campaigns to target

2010				Diet difficult to measure accurately in the study, unable to measure socioenvironmental factors that influence development	incidence of Impaired Glucose Tollerance and T2DM. Smoking and physical activity behaviours were also related to this	modifiable lifestyle behaviours and high-risk groups for T2DM
E.D Janus Et al. 2007	Rural Victoria & South Australia, AU	~52.4% female	806 adults aged 25-74	Small sample population	Higher prevalence of general obesity in men; higher prevalence of central obesity in women	Urgent action to address unhealthy lifestyle and diet habits
D Simmons Et al 2005	Rural Victoria, AU	~56% female	1451 adult residents of Goulburn Valley	Dietary data did not measure energy intake, Nutritional items limited, No question regarding high calorie drinks. Used Victorian Health Survey questions for intake	Fast food consumption was not associated with obesity, although those who consumed no take-away had a smaller Waist Circumference. Physical activity identified as linking to obesity	Implementation of strategies to increase physical activity. Investigation into food supply/ dietary intake. Deeper look into dietary habits

NB: Literature search used the following search terms “diet”, “intake”, “food”, “fruit” and “vegetables”, alongside “obesity”, “BMI”, “waist circumference” and “rural Australia”. Studies were assessed and excluded if they targeted a specific gender, ethnicity, or cultural background for the purpose of consistency in cohort comparison.
